# A Feasibility Study of Deep Learning-Based Auto-Segmentation Directly Used in VMAT Planning Design and Optimization for Cervical Cancer

**DOI:** 10.3389/fonc.2022.908903

**Published:** 2022-06-01

**Authors:** Along Chen, Fei Chen, Xiaofang Li, Yazhi Zhang, Li Chen, Lixin Chen, Jinhan Zhu

**Affiliations:** ^1^ Department of Radiation Oncology, State Key Laboratory of Oncology in South China, Collaborative Innovation Center for Cancer Medicine, Sun Yat-sen University Cancer Center, Guangzhou, China; ^2^ School of Biomedical Engineering, Guangzhou Xinhua University, Guangzhou, China; ^3^ Department of Radiation Oncology, The Second Affiliated Hospital of Zunyi Medical University, Zunyi, China; ^4^ Department of Oncology and Hematology, The Six People’s Hospital of Huizhou City, Huiyang Hospital Affiliated to Southern Medical University, Huizhou, China

**Keywords:** deep learning, automatic segmentation, dosimetric differences, geometric accuracy, cervical cancer

## Abstract

**Purpose:**

To investigate the dosimetric impact on target volumes and organs at risk (OARs) when unmodified auto-segmented OAR contours are directly used in the design of treatment plans.

**Materials and Methods:**

A total of 127 patients with cervical cancer were collected for retrospective analysis, including 105 patients in the training set and 22 patients in the testing set. The 3D U-net architecture was used for model training and auto-segmentation of nine types of organs at risk. The auto-segmented and manually segmented organ contours were used for treatment plan optimization to obtain the AS-VMAT (automatic segmentations VMAT) plan and the MS-VMAT (manual segmentations VMAT) plan, respectively. Geometric accuracy between the manual and predicted contours were evaluated using the Dice similarity coefficient (DSC), mean distance-to-agreement (MDA), and Hausdorff distance (HD). The dose volume histogram (DVH) and the gamma passing rate were used to identify the dose differences between the AS-VMAT plan and the MS-VMAT plan.

**Results:**

Average DSC, MDA and HD_95_ across all OARs were 0.82–0.96, 0.45–3.21 mm, and 2.30–17.31 mm on the testing set, respectively. The D_99%_ in the rectum and the Dmean in the spinal cord were 6.04 Gy (P = 0.037) and 0.54 Gy (P = 0.026) higher, respectively, in the AS-VMAT plans than in the MS-VMAT plans. The V_20_, V_30_, and V_40_ in the rectum increased by 1.35% (P = 0.027), 1.73% (P = 0.021), and 1.96% (P = 0.008), respectively, whereas the V_10_ in the spinal cord increased by 1.93% (P = 0.011). The differences in other dosimetry parameters were not statistically significant. The gamma passing rates in the clinical target volume (CTV) were 92.72% and 98.77%, respectively, using the 2%/2 mm and 3%/3 mm criteria, which satisfied the clinical requirements.

**Conclusions:**

The dose distributions of target volumes were unaffected when auto-segmented organ contours were used in the design of treatment plans, whereas the impact of automated segmentation on the doses to OARs was complicated. We suggest that the auto-segmented contours of tissues in close proximity to the target volume need to be carefully checked and corrected when necessary.

## 1 Introduction

In radiotherapy, automatic delineation of normal tissues based on deep learning techniques is an increasingly mature technique, and the automatic delineation of target volumes has been explored in successive multicentre clinical application studies. The convolutional neural network (CNN) is superior to most other algorithms in the segmentation of medical images (1), and, as a result, it is often used for the automatic delineation of normal tissues and target volumes (2–5) on computed tomography (CT) images of the head and neck (6–8), chest (9), abdomen (10, 11), and pelvic cavity (5, 12–15), among others.

Radiotherapy is an effective treatment for cervical cancer (16, 17), and delivery of precision radiotherapy requires accurate contouring of each organ on the patient’s CT images. Manual segmentation of normal tissues depends on the experience and ability of the imaging radiologist (18, 19) and has a low efficiency. The poor contrast of pelvic soft tissues on CT images also presents challenges for radiologists. With the rapid development of image segmentation techniques, CNN-based automated organ contouring on CT images has become increasingly popular for patients with cervical cancer. Liu et al. (20) used the improved U-Net model to automatically segment cervical cancer organs at risk (OARs), and the model prediction was highly consistent with the OARs delineated by radiation oncologists. Ju et al. (21) innovatively integrated the Dense Net model with the V-Net model, enabling accurate, efficient, and automatic delineation of six OARs on CT images. Qualitative and quantitative studies conducted by Rhee et al. (5) showed that the auto-contouring tool based on CNN can be used to generate the segmentation of OARs and clinical target volume (CTV) for patients with cervical cancer and achieve clinically acceptable delineation results.

Despite these encouraging results, many challenges remain to be overcome before auto-segmentation methods can be applied in clinical practice. First, patients with cervical cancer are treated in supine or prone positions, and no study has examined whether different patient positions affect automatic delineations of normal tissues. Second, there remains room for improvement in the accuracy of automatic soft tissue segmentation, such as in colons and rectums. More importantly, existing assessments of accuracy in automated normal tissue segmentation are limited to the comparison of geometric accuracy, and few studies have focused on their relevant dosimetric impact. However, a model successfully segments the OARs in geometry is not sufficient to confirm its reliability for clinical application. Fung et al. (22) and Zhu et al. (23) introduced their dosimetric evaluation methods about dose impact between manually and automatically segmented OARs. Vinod et al. (24) believed that it is important to quantify the degree of uncertainty in volume segmentation, but the resulting impact on dosimetry and clinical significance is a more relevant endpoint.

Patients with cervical cancer with different therapeutic positions were included in this study for model training. We then performed automatic delineation of nine types of normal tissues and evaluated its geometric accuracy. On this basis, we discussed the impact of unmodified auto-contouring of tissue structures on the design and optimization of treatment plans. We attempted to use experimental data to investigate the following: 1) whether the dose distribution inside the clinical target volume is affected, and in the case of dose deviations, whether these deviations are within a clinically acceptable range; and 2) whether dose deviations to organs at risk are clinically acceptable.

## 2 Materials and Methods

### 2.1 Case Selection

This study included a total of 127 patients with cervical cancer who received radiotherapy at Sun Yat-sen University Cancer Centre between December 2020 and August 2021, including 65 patients in the supine position and 62 patients in the prone position. None of the included patients underwent intestinal tract modification surgery. The images were obtained using a Philips large-aperture CT simulation scanner (Philips Brilliance Big Bore, Netherlands) at 140 keV voltage and a 3-mm slice thickness. The size of images for each slice was 512 × 512 and the number of slices ranged between 140 and 205.

Three clinicians used the Monaco (V5.11) treatment planning system to manually segment bone structures (including the left femoral head, the right femoral head, and the pelvis) as well as tissues and organs (including the spinal cord, the left and right kidneys, the bladder, the rectum, and the colon) from the patient’s CT images. Each organ at risk was segmented in strict accordance with the requirements in the radiation therapy oncology group (25) guidelines and the delineation results were reviewed and modified by senior radiation therapists.

### 2.2 Data Pre-Processing

The 105 sets of CT images obtained were used for model training, including 52 sets obtained in the supine position and 53 sets obtained in the prone position. To increase the training sample size, the CT images were cropped into sub-images 100 × 100 × 100 in size, with random positions selected in the whole body range as the starting points. In addition, 22 sets of CT images were selected for model testing, including 13 sets obtained in the supine position and 9 sets obtained in the prone position.

To highlight the soft tissues, bones, and bladders in the images, we also added 3 types of images processed with different window widths and window levels to the original input sub-images, including soft tissue images: window width = 400, window level = 40; bone images: window width = 1000, window level = 400; bladder images: window width = 250, window level = 50. Hence, the input to the training model was: 4 × 100 × 100 × 100. All input images were normalised to the range of 0–1.

### 2.3 Model Training

**Figure 1:**
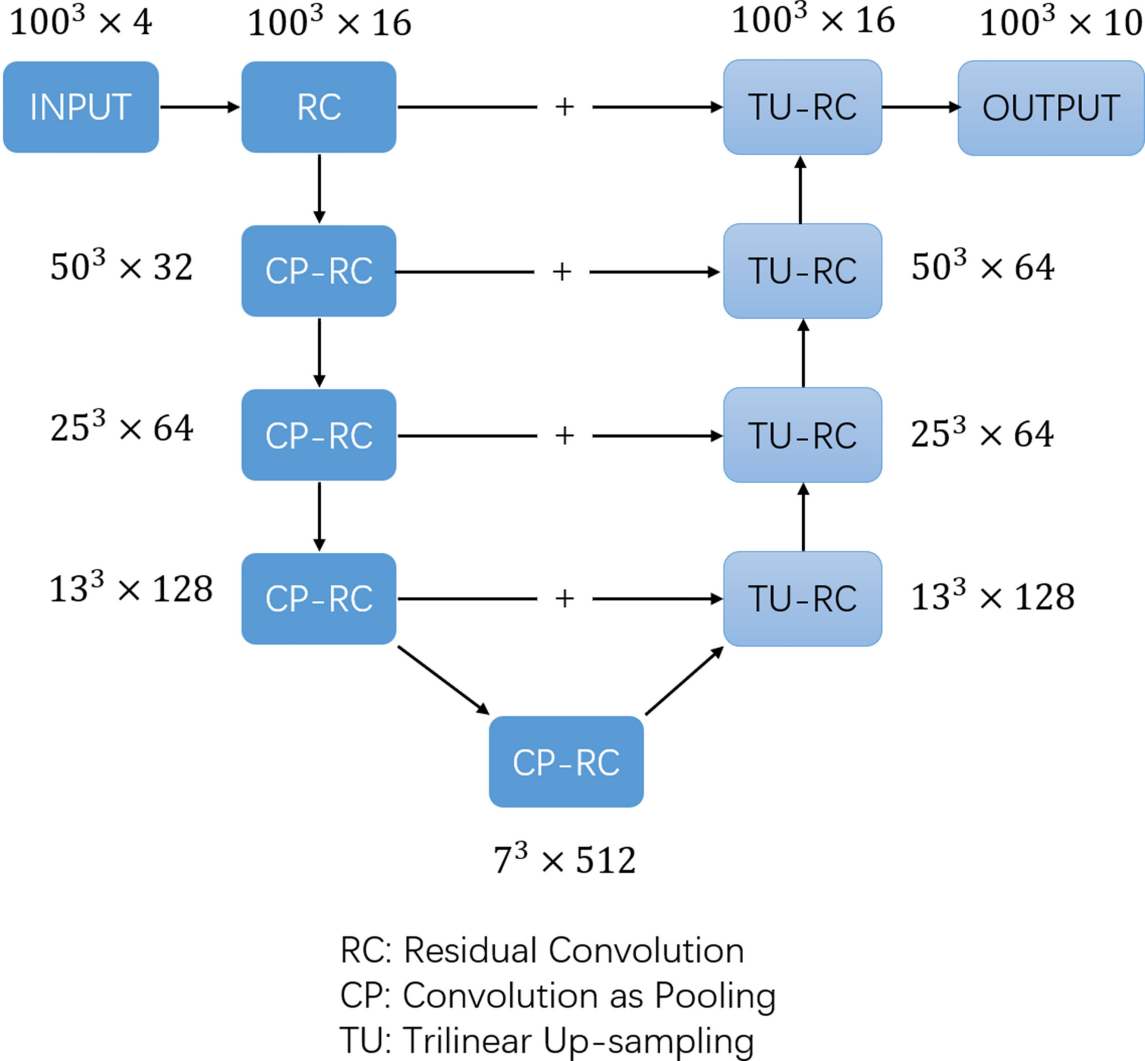
The structure of 3D U-net network.

The training labels were filled with one-hot images containing ten channels according to the manually segmented structures. The one-hot images were binary class matrices which have zeros everywhere except where the index of channel matches the corresponding value of the class number, in which case it will be 1. The 1^st^ channel represented the undelineated areas; the 2^nd^ channel was marked as the bladder (bladder); the 3^rd^ channel was marked as the left femoral head (femoral_ joint L); the 4^th^ channel was marked as the right femoral head (femoral_ joint R); the 5^th^ channel was marked as the rectum (rectum); the 6^th^ channel was marked as the colon (colon); the 7^th^ channel was marked as the left kidney (Kidney-L); the 8^th^ channel was marked as the right kidney (Kidney-R); the 9^th^ channel was marked as the pelvic bone (PelvicBone); and the 10^th^ channel was marked as the spinal cord (SpinalCord). The dice similarity coefficient (DSC) is commonly used to measure the overlap of two structures (26, 27), and was adopted as the loss function, while the AdamW (28, 29) optimizer was used to train the CNN network. The batch size was set to 2 in the training algorithm and the learning rate was set using the OneCycleLR learning rate scheduler (30), with the maximum learning rate set to 0.01 and the minimum learning rate set to 4e^-8^. Cosine annealing was adopted to schedule the learning rate and the step size was set to per sample. The model was trained for a total of 30 epochs and the model parameters were updated based on the minimum loss value of the evaluation set. The 3D U-net architecture (**Figure 1**) used in previous studies (31) was adopted in the model and a 1 × 1 × 1 convolution kernel was utilised in the last layer, with SoftMax as the activation function. The number of image layers with eigenvalues was reduced to 10 before data output.

### 2.4 Assessment Indicators

#### 2.4.1 Assessment of Geometrical Differences

The manually segmented organ contours served as “the golden standard” and the auto-segmentation results were compared with the manual delineation results to assess the accuracy. The assessment indicators include the DSC, the Hausdorff Distance (HD), the 95th percentile of the HD, and the Mean Distance to Agreement (MDA) (32). The commercial software MIM (V6.9, MIM Software Inc., Cleveland, OH, USA) and 3D Slicer (V4.8.1) were used to identify and evaluate the geometrical differences between the automated and manual segmentation results.

#### 2.4.2 Evaluation of Dose Differences

To evaluate the impact of geometrical differences between automated and manual segmentation on the dosimetric parameters in treatment plans, we selected 22 patients from the testing set and performed optimization procedures with auto-segmented organ contours to obtain new treatment plans (automatic segmentations VMAT, AS-VMAT); this was performed without changing the parameter setting of the cost function for treatment plan optimization and other optimization parameters. These new treatment plans were compared to those optimised using manually segmented organ contours (manual segmentations VMAT, MS-VMAT) to identify the differences in dose to OARs. The dose differences to OARs were evaluated with the following parameters: D_1%_, D_2%_, Dmean, D_98%_, D_99%_, V_10_, V_20_, V_30_, V_40_, and V_50_. Two assessment methods were adopted, and the specific compared items are shown in **Table 1**.

**Table 1:** Specific compared items in the evaluation of dose differences to organs at risk (two evaluation methods).

Evaluation methods	ASAP vs. MSMP		MSAP vs. MSMP	
Structures	Automatic segmentations	Manual segmentations	Manual segmentations	Manual segmentations
Plans	AS-VMAT plans	MS-VMAT plans	AS-VMAT plans	MS-VMAT plans

ASAP, Automatic Segmentation in AS-VMAT Plan; MSAP, Manual Segmentation in AS-VMAT Plan; MSMP, Manual Segmentation in MS-VMAT Plan; AS-VMAT, automatic segmentations VMAT; MS-VMAT, manual segmentations VMAT.

The SPSS25.0 software was used for statistical analysis, and the data were first tested for conformance with a normal distribution. The paired t-test was performed on the normally distributed data, while the Wilcoxon signed rank test was performed on the data that did not conform to a normal distribution. A P-value < 0.05 was considered to be statistically significant.

To evaluate the sensitivity in the detection of dosimetry differences, the dosimetry results for manually segmented organ contours in the MS-VMAT plans were used to define the 95% confidence intervals and the cut-off values of the parameters evaluating the dosimetry differences in OARs. The number of cases in which the dosimetry results for the auto-segmented organ contours/manually segmented organ contours were outside the confidence interval in the AS-VMAT plan was calculated. The SPSS 25.0 statistical software was used to calculate the 95% confidence interval of the evaluation parameters (Formula 1).


(1) $$CL_{M_{C}}=mean\pm 1.96\sigma$$

where *mean* represents the mean value of the evaluation parameter; *σ* denotes the corresponding standard deviation; and *CL* denotes the confidence interval.

To evaluate the CTV coverage, the percent coverage of CTV V_42.75_ and CTV V_45_ in the AS-VMAT plans and the MS-VMAT plans was evaluated. The dose distributions in the AS-VMAT plans were compared to those in the MS-VMAT plans to evaluate the differences in CTV gamma passing rates (2%/2 mm and 3%/3 mm criteria). The threshold dose was set at 95% of the prescription dose, because in clinical practice, more attention is paid to tumour control and normal tissue toxicity in high-dose areas (33).

## 3 RESULTS

### 3.1 Results of the Evaluation of Geometrical Differences

**Figure 2:**
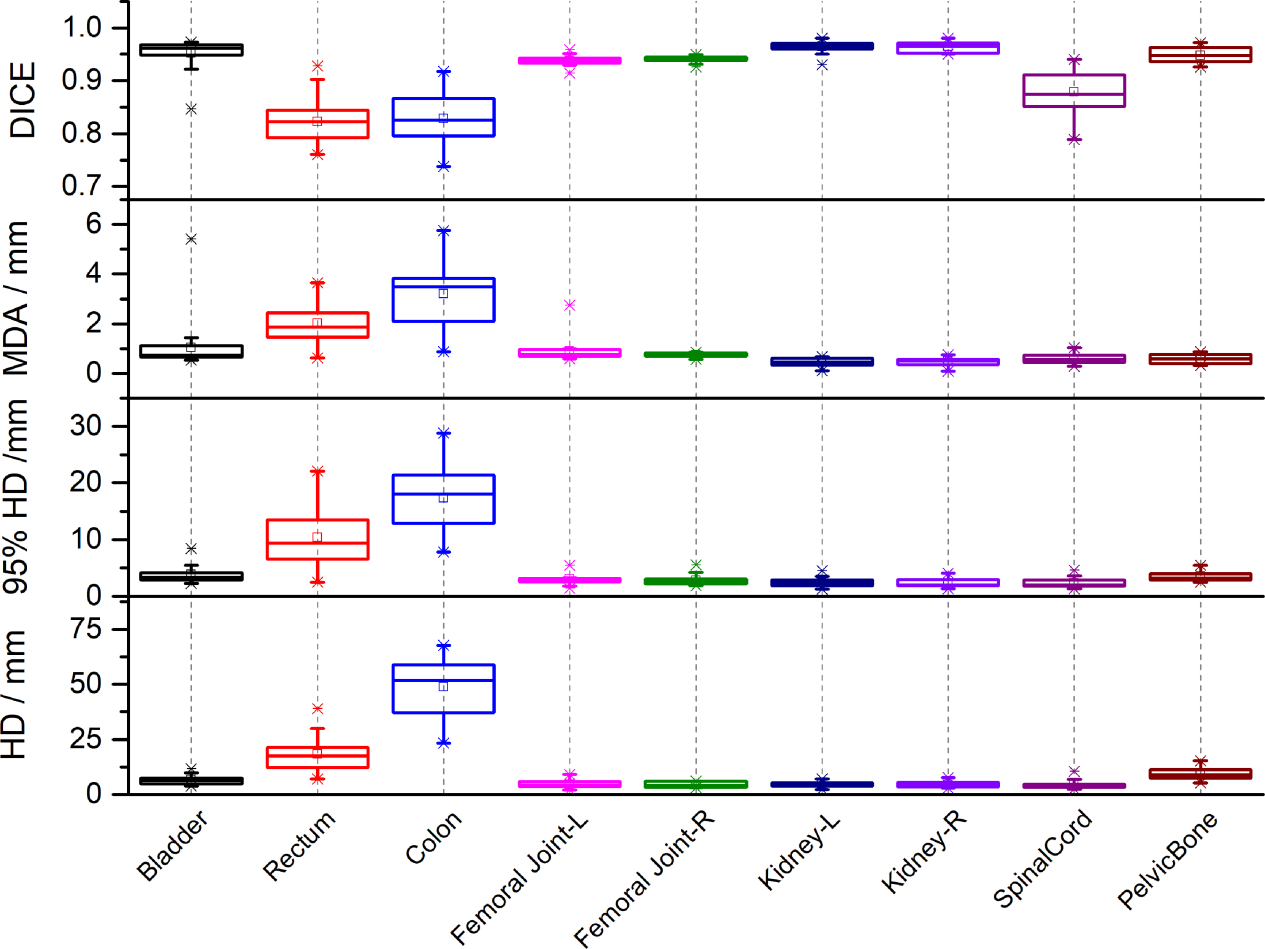
The Dice similarity coefficients (DSC), Mean Distance to Agreement (MDA), Hausdorff Distance (HD) and 95th-percentile of the HD between automated segmentation and manual segmentation for each organ at risk in the testing set.

**Figure 2** lists the DSC, MDA, HD and HD_95_ between manual segmentation and automated segmentation for each organ at risk in the testing set. The mean DSC (range) between manual segmentation and automated segmentation for all organs at risk was 0.91 (0.82–0.96). The automated segmentation results were highly similar to those of the manual segmentation results in the bladder, the femoral head, the kidney, and the pelvic bone, with a mean DSC of > 0.94. The mean DSCs in the colon and the rectum were 0.82 and 0.83, respectively. The mean MDA, HD and HD_95_ (range) between manual segmentation and automated segmentation for all organs at risk were 1.17 mm (0.45–3.21 mm), 11.73 mm (4.34–48.72 mm) and 5.32 (2.30–17.31 mm), respectively. The MDA and the HD_95_ were the largest in the colon, with mean values of 3.21 ± 1.26 mm and 48.72 ± 12.60 mm, respectively.

### 3.2 Results of the Evaluation of Dose Differences

**Figure 3:**
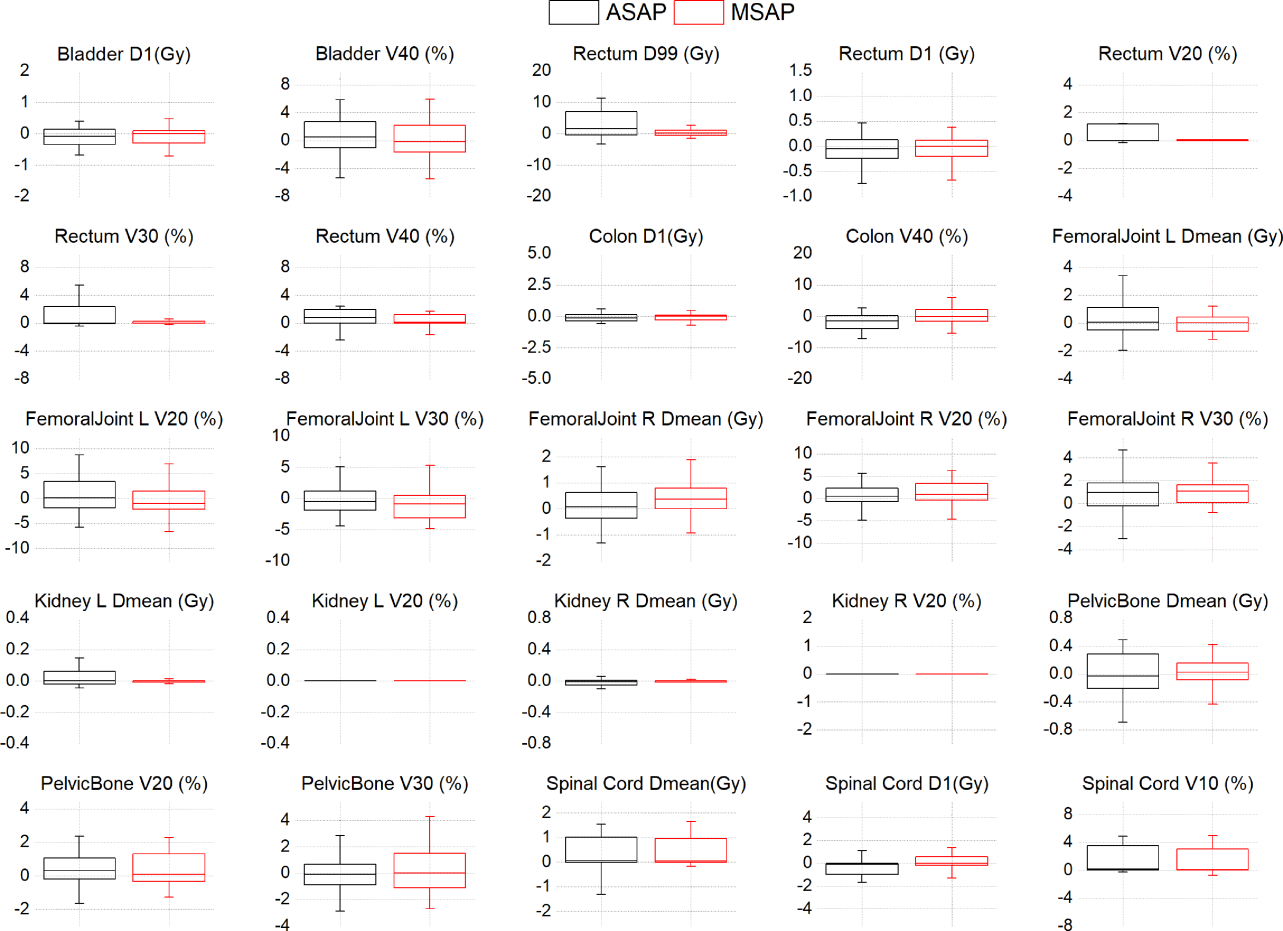
The dose differences of DVH parameters between the AS-VMAT plans and the MS-VMAT plans of 22 patients.The black box represents the ASAP vs. MSMP results, and the red box represents the MSAP vs. MSMP results. ASAP, Automatic Segmentation in AS-VMAT Plan; MSAP, Manual Segmentation in AS-VMAT Plan; MSMP, Manual Segmentation in MS-VMAT Plan.

**Figure 3**; **Supplementary Table A** shows that compared to the dose distribution within manually segmented organ contours in the MS-VMAT plans of 22 patients, the D_99%_ within the auto-segmented rectum contours and the Dmean within the auto-segmented spinal cord contours in the AS-VMAT plans were higher by 6.04 Gy (P = 0.037) and 0.54 Gy (P = 0.026), respectively. The V_20_, V_30_, and V_40_ in the rectum increased by 1.35% (P = 0.027), 1.73% (P = 0.021), and 1.96% (P = 0.008), respectively, whereas the V_10_ in the spinal cord increased by 1.93% (P = 0.011). The differences in other dosimetry parameters were not statistically significant.

Based on the dose distribution within the manually segmented organ contours, the dose differences between the AS-VMAT plans and the MS-VMAT plans were relatively small. The D_99%_ in the rectum was higher by 0.64 Gy (P = 0.292), with no significant differences. The Dmean in the spinal cord was higher by 0.53 Gy (P = 0.044). The V_40_ in the rectum increased by 1.00% (P = 0.034), while the V_10_ and V_20_ in the spinal cord increased by 1.76% (P = 0.015) and 1.59% (P = 0.015), respectively. The differences in other dosimetry parameters were not statistically significant.

The AS-VMAT plans of 22 cases were used to evaluate the sensitivity in the detection of dosimetry differences. Among the results for both automatically and manually segmented organ contours, the dosimetry results outside the confidence interval for the bladder (D_1%,_ D_2%_ and V_40_) and the rectum (D_1%_ and D_2%_) were found in 2 cases each. Among the results for auto-segmented organ contours, the dosimetry results outside the confidence interval for the rectum (V_40_), the colon (D_1%_ and D_2%_), the right femoral head (V_30_), the left kidney (Dmean), and the pelvis (Dmean and V_30_) were found in 1 case each. Among the results for manually segmented organ contours, the dosimetry results outside the confidence interval for the colon (D_1%_ and D_2%_), the right femoral head (V_30_), the left and right kidneys (Dmean), and the pelvis (Dmean) were found in 1 case each, with a percentage outside the confidence interval of < 10%. No dosimetry results were outside the confidence interval for other evaluation parameters in any of the cases.

**Figure 4:**
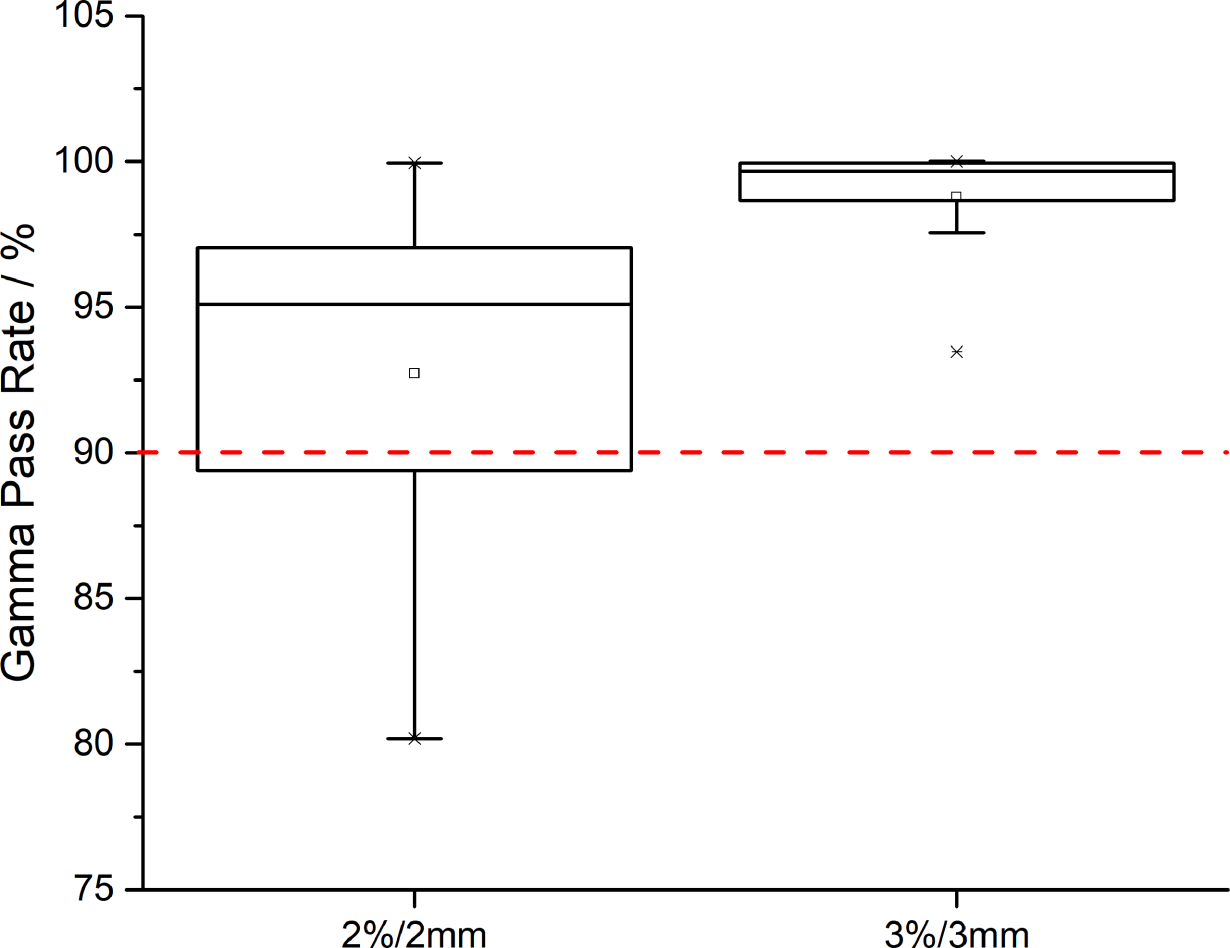
The correspondence between the AS-VMAT plans and the MS-VMAT plans in terms of gamma passing rates in clinical target volume (CTV). The red dotted line denotes a gamma passing rate of 90%. AS-VMAT, automatic segmentations VMAT; MS-VMAT, manual segmentations VMAT.

Regarding the evaluation of CTV coverage, in the AS-VMAT plans, the percent coverage of CTV V42.75 and CTV V45 was 99.86% ± 0.33% and 99.47% ± 1.67%, respectively, and the corresponding percent coverage in the MS-VMAT plans was 99.77% ± 0.75% and 99.53% ± 0.98%, respectively. The mean percent coverage of CTV V42.75 and the mean percent coverage of CTV V45 were higher by 0.09% (P = 0.453) and lower by 0.06% (P = 0.109), respectively in the AS-VMAT plans compared to the MS-VMAT plans. **Figure 4** shows the correspondence between the AS-VMAT plans and the MS-VMAT plans in terms of gamma passing rates in CTV. The mean gamma passing rates were 92.72% and 98.77%, respectively using the 2%/2 mm and 3%/3 mm criteria, which satisfied the clinical requirements.

**Figure 5:**
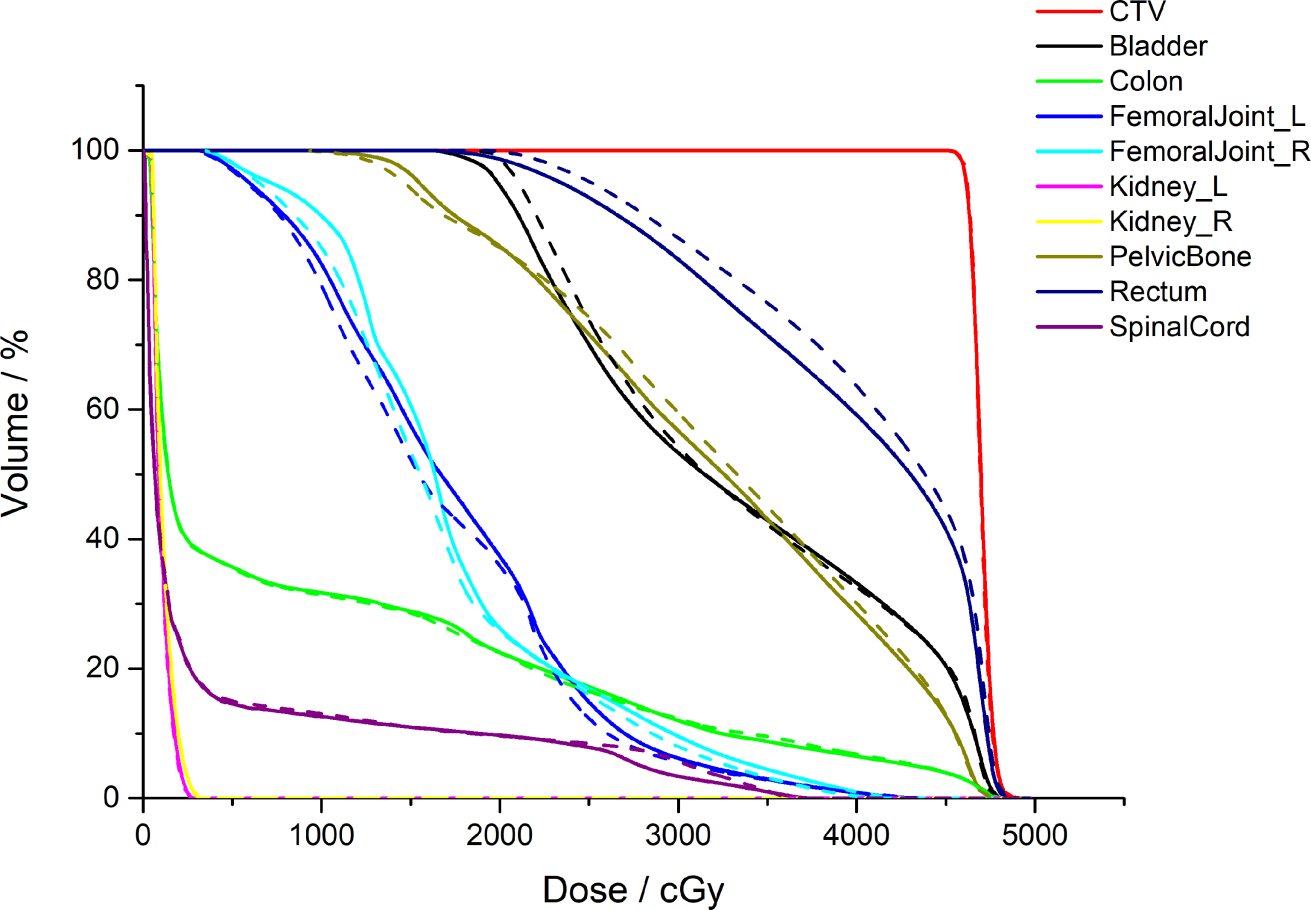
Comparison of the clinical target volume (CTV) and normal tissue DVHs for one patient with cervical cancer. The dotted lines denote the treatment plans optimised by auto-segmented organ contours (AS-VMAT), whereas the solid lines denote the treatment plans optimised by manually segmented organ contours (MS-VMAT). AS-VMAT, automatic segmentations VMAT; MS-VMAT, manual segmentations VMAT.

**Figure 5** shows a comparison between the AS-VMAT plans and the MS-VMAT plans in terms of CTV and normal tissue DVHs for one patient with cervical cancer. There was no significant difference in the dose to CTV between the VMAT treatment plans optimised with manually segmented organ contours and those optimised with auto-segmented organ contours. There were insignificant dose deviations in normal tissue volume receiving < 30 Gy, such as in the bladder, the rectum, the pelvis, and the femoral head; there were dose deviations in the rectal volume receiving 30Gy–40Gy; and there was basically no dose difference to other normal tissues.

## 4 DISCUSSION

The convolutional neural network algorithm based on multi-layer supervised learning features good fault-tolerance, and strong adaptability and weight-sharing (13, 14, 34, 35). The results generated by the trained model are reliable and applicable in clinical practice. We used the 3D U-net model for the auto-segmentation of nine types of normal tissues. The results suggested high geometric accuracy of automatic segmentation for the bladder, the femoral head, the pelvis, and the kidney, with a Dice value of > 0.94, which is consistent with, or even better than the results reported previously. The main reasons for this include the high density of bone structures (the pelvis and the femoral head) and strong tissue contrast. Indeed, the fluid-filled bladder can be easily distinguished from adjacent soft tissues, while there is a clear-cut anatomical position of the kidneys in the human body.

Relatively speaking, auto-segmentation of intestinal tissues, such as the colon and rectum, has a lower accuracy. Our results showed that auto-segmentation of the rectum and the colon featured a larger HD and a Dice value of 0.82 and 0.83 (< 0.9), respectively. Compared to previous results, Men et al. (14) reported a Dice value of 0.618 for the segmentation of the colon using a deep dilated convolutional neural network (DDCNN), which is lower than our study results; Rhee et al. (5) reported a Dice value of 0.80 for the segmentation of the rectum based on the CNN model, which is roughly equivalent to our study results; and Ju et al. (21) reported a Dice value of 0.87 for the segmentation of the rectum using an innovative fused model Dense V-Network, which is similar to our results. Generally, the Dice value for the segmentation of intestinal tissues can reach approximately 0.8 if proper neural networks and learning models are used (including 3D Unet and Dense-V-Network).

Auto-segmentation of intestinal tissues has a lower accuracy largely because the intestinal tract is a soft tissue with low-contrast image performance in CT images. For example, in terms of the rectum, the lower boundary of the rectum is connected to the anal canal and the boundary between the anal canal and the rectum is unclear on CT images, which makes it challenging to accurately identify the position of the lower boundary. In addition, the upper boundary of the rectum is connected to the sigmoid colon with an anatomical boundary between the rectum and the sigmoid colon, but this boundary is difficult to accurately identify *via* imaging. In terms of the colon, as we included patients treated in both the prone and the supine positions, and given that in some patients in the prone position, the position of the colon was pushed upward, the colon was not well distinguished from the pulmonary cavity and the aerated gastric body during auto-segmentation, resulting in segmentation failure. In addition, the accuracy of auto-segmentation of intestinal tissues is affected by the amount of faeces and gas in the intestines, which is a common problem with other automatic segmentation models when the intestinal organs are segmented. The single learning model that we used is well suited to patients in different therapeutic positions, and there is no need to construct different learning models for supine positions and prone positions independently, which is why we included patients treated in different body positions.

We sought to determine whether we could directly use the unmodified normal tissue contours in the design of treatment plans given that the auto-segmented normal tissue contours are highly similar to manual segmentation results, and whether the dosimetry results in the optimised treatment plan satisfy the clinical requirements. As can be seen from the study results, irrespective of whether the treatment plans were optimised by auto-segmented or manually segmented normal tissue contours, the dose differences in the target volumes were relatively small (i.e., the doses to CTV were highly consistent). In this study, the gamma passing rate was adopted for quality assurance of treatment plans. Even when using the strict 2%/2 mm criterion, the gamma passing rates were > 90%, indicating that the dosimetry results are acceptable for clinical use.

**Figure 6:**
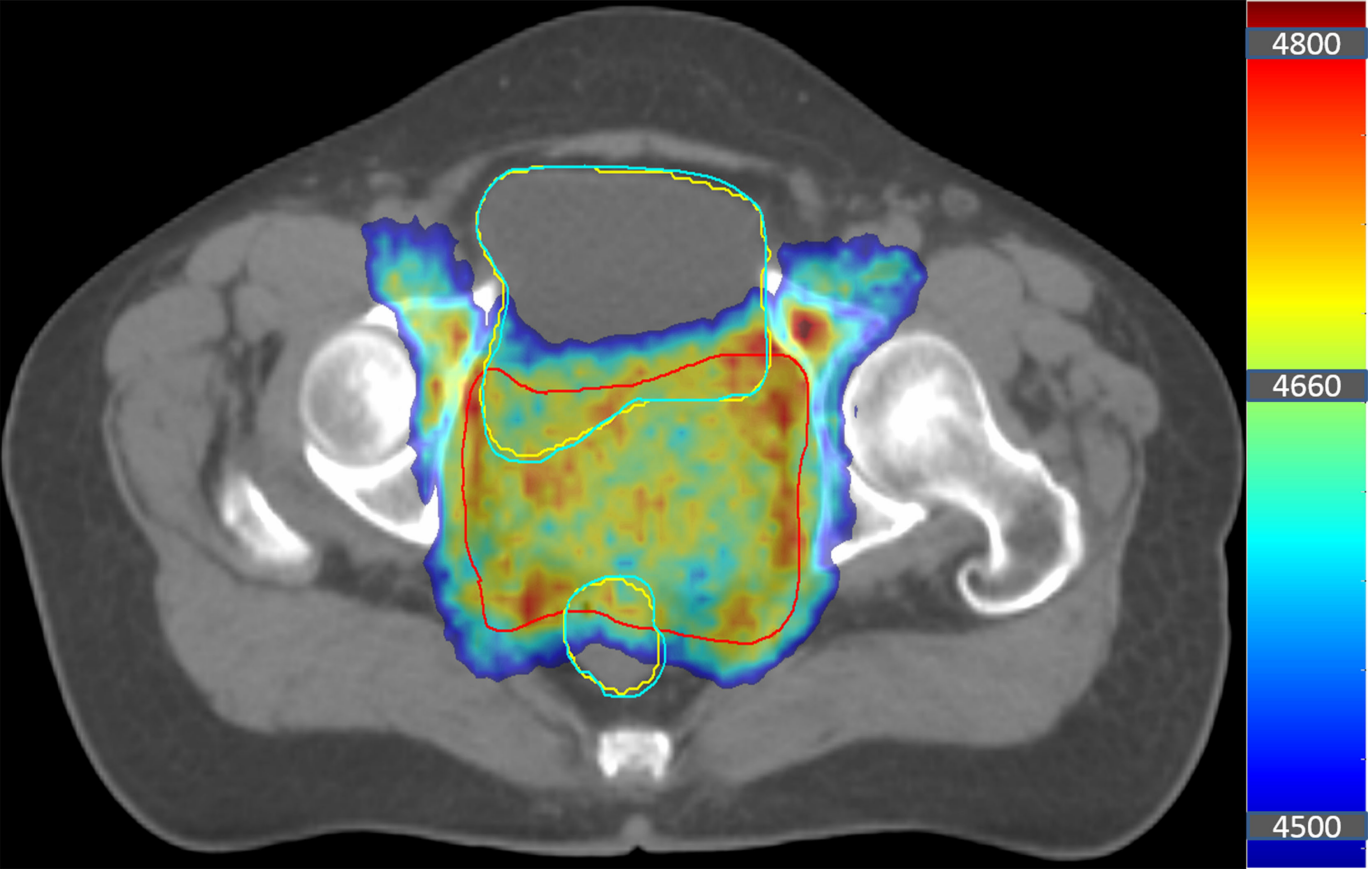
Diagram showing the position of the target volume, the bladder, and the rectum in a patient with cervical cancer. The coloured area denotes the target volume receiving > 45 Gy. The area marked with the red solid line is the clinical target volume (CTV), the blue solid lines denote the manually segmented contours, and the yellow solid lines denote the auto-segmented contours.

As for the dose differences of automated segmentation of organs at risk, the situation is more complex and the organs at risk can be divided into three types:

1) The first type of organs, including the left and right femoral heads and the left and right kidneys, were located at a distance from the target volume, and automated segmentation of their contours was accurate. When these auto-segmented normal tissue contours were directly used for the design of treatment plans, the generated dosimetry parameters were not significantly different from those of the MS-VMAT plans. The spinal cord is an exception; although the spinal cord was located at a distance from the target volume and the auto-segmented contours were highly similar to those of manually segmented contours, the differences between the two sets of plans in terms of Dmean and V_10_ in the spinal cord were statistically significant due to the excessively small volume of the spinal cord (P < 0.05). A common problem in cord segmentation was the length of cord contoured which adversely affected Dice and Dmean but had no clinical significance. Specifically, the absolute dose difference in Dmean was < 0.54 Gy and the volume difference to the V_10_ was < 2%. Hence, these dosimetry results differences appear to be clinically acceptable, and the spinal cord is still classified as a type I organ at risk.

2) The second type of organs, including the pelvis and the colon, overlapped with the target volume on some CT slices. The volume of the overlap region accounted for a relatively small percentage of the total organ volume, so the geometrical differences in automated segmentation results did not result in large dose deviations and did not affect the dosimetry results in clinical evaluation. The obvious errors in automated organ segmentation need to be addressed and corrected, especially errors in areas close to the target volume. The abovementioned organs are classified as type II organs at risk.

3) The third type of organs, including the rectum and the bladder, were close to the target volume. The differences between the MS-VMAT plans and the AS-VMAT plans in terms of the D_98%_, D_99%_, V20, V_30_, and V_40_ in the rectum were statistically significant (P < 0.05). In addition, dosimetry results outside the confidence interval for the bladder (D_1%,_ D_2%_ and V_40_) and the rectum (D_1%_ and D_2%_) were found in 2 cases each. This may be because the rectum and the bladder were close to the CTV, even overlapping in some regions (as shown in **Figure 6**). Hence, the geometrical differences in automated segmentation results had a significant impact on the dose received by high-dose areas. Meanwhile, the dosimetry results were more sensitive to the geometric accuracy of automated contouring due to the relatively small volume of the rectum. Therefore, auto-segmented organ contours need to be carefully checked, with the errors corrected. The abovementioned organs are classified as type III organs at risk.

Moreover, another factor contributing to the difference in planned dose lies in the treatment planning system. During the course of the study, we found that after the treatment plan was optimised twice under identical optimization conditions for the same patient (same structures and CT images) in the Monaco system, the generated sequences of the sub-fields and the positions of the leaves were not entirely consistent, which resulted in significant differences in the dose distribution within low-dose areas.

We attempted to segment the normal tissues in patients with cervical cancer using deep learning techniques. In addition, we attempted to analyse which tissues received significantly different doses when automated segmentation results with high geometric accuracy were directly used in the design of treatment plans. Based on the results, we classified the auto-segmented normal tissues into three types. The auto-segmentation results for some tissues need to be carefully checked and corrected, while the auto-segmented contours of other tissues can be almost left unmodified, thereby saving clinicians a significant amount of time (an important objective of this study). A similar finding was reported by Vaasen et al. (36), that most OARs can be left unedited except under certain circumstances where they were close to the planning target volume. However, this study still has its limitations. First, the size of the samples from the testing set was too small to accurately evaluate the dose differences and larger sample sizes will provide more statistically significant results. Second, the analysed patients were collected from the same medical centre and no multicentre comparison was performed. Conclusions based on multicentre studies would be more objective and compelling.

## 5 CONCLUSIONS

The 3D U-net model can be used for accurate, efficient, and automated segmentation of organs at risk in patients with cervical cancer. When auto-segmented organ contours were used in the design of treatment plans, the dose distributions of target volumes were not affected, whereas the impact of automated segmentation on the doses to organs at risk was complicated. We suggest that the auto-segmented contours of tissues in close proximity to the target volume need to be carefully checked and corrected when necessary, while auto-segmented contours of tissues at a distance from the target volume can be left largely unmodified.

## Data Availability Statement

The datasets presented in this study can be found in online repositories. The names of the repository/repositories and accession number(s) can be found below: https://www.researchdata.org.cn/RDDA2022345612.

## Author Contributions

AC and FC are responsible for data analysis and paper writing. XL, YZ, and LC are responsible for manually delineating the organs, and for data collection. JZ is responsible for program design and model training. JZ and LXC are responsible for are responsible for research strategy design and paper revision. All authors contributed to the article and approved the submitted version.

## Funding

This study was jointly supported by the National Natural Science Foundation of China (No. 12005315) and the National Natural Science Foundation of China (No.12075329).

## Conflict of Interest

The authors declare that the research was conducted in the absence of any commercial or financial relationships that could be construed as a potential conflict of interest.

## Publisher’s Note

All claims expressed in this article are solely those of the authors and do not necessarily represent those of their affiliated organizations, or those of the publisher, the editors and the reviewers. Any product that may be evaluated in this article, or claim that may be made by its manufacturer, is not guaranteed or endorsed by the publisher.
