# Supplementary material for: A Feasibility Study of Deep Learning-Based Auto-Segmentation Directly Used in VMAT Planning Design and Optimization for Cervical Cancer
Source: Front Oncol. 2022 Jun 1;12:908903. doi: 10.3389/fonc.2022.908903 (PMC9198405; doi:10.3389/fonc.2022.908903)
Supplement: Supplementary file 1 [file Table_1.docx]

**Supplementary Table A:** Differences in the dose distribution within automated/manually segmented organ contours between the AS-VMAT plans and the MS-VMAT plans

| **OARs** | **Dose Parameters** | | | | | | | | | |
| --- | --- | --- | --- | --- | --- | --- | --- | --- | --- | --- |
|  | D98%（Gy） | D99%（Gy） | Dmean（Gy） | D1%（Gy） | D2%（Gy） | V10（%） | V20（%） | V30（%） | V40（%） | V50（%） |
| ASAP VS. MSMP | | | | | | | | | | |
| Bladder | 0.32±1.55 | 0.25±1.66 | 0.06±0.47 | －0.27±0.95 | －0.26±0.73 | －0.04±0.15 | 0.11±1.38 | 0.40±1.86 | 0.64±3.43 | －0.24±0.70 |
| Rectum | 5.19±9.57 | 6.04±9.68 | 0.48±1.32 | －0.04±0.37 | －0.04±0.35 | 0.69±2.31 | 1.35±3.12 | 1.73±3.62 | 1.96±3.71 | －0.46±2.79 |
| Colon | 0.30±1.48 | 0.32±1.40 | －0.46±2.89 | 1.12±4.26 | 1.03±4.59 | －1.01±6.40 | －1.32±6.46 | －1.07±6.48 | －0.75±5.82 | 0.47±1.74 |
| Femoral Head L | －0.09±0.60 | －0.09±0.66 | 0.20±1.13 | －0.18±1.01 | 0.10±1.19 | 3.27±8.90 | 0.22±4.66 | －0.63±3.53 | －0.05±0.98 | 0.00±0.00 |
| Femoral Head R | 0.30±1.78 | 0.29±1.92 | 0.37±1.09 | 0.50±1.48 | 0.63±1.60 | 1.47±8.37 | 1.96±7.07 | 0.88±1.79 | 0.03±0.77 | 0.00±0.00 |
| Kidney L | －0.02±0.08 | －0.01±0.07 | －0.06±0.26 | －0.29±0.65 | －0.18±0.43 | 0.03±0.48 | －0.05±0.14 | －0.03±0.11 | 0.00±0.00 | 0.00±0.00 |
| Kidney R | 0.06±0.26 | 0.06±0.23 | －0.09±0.24 | 0.22±1.22 | 0.17±0.96 | －0.31±0.64 | －0.07±0.61 | －0.02±0.09 | 0.00±0.00 | 0.00±0.00 |
| Pelvic Bone | 0.12±0.52 | 0.11±0.54 | －0.03±0.34 | －0.01±0.44 | 0.03±0.25 | 0.14±0.39 | 0.38±0.95 | 0.10±1.63 | －0.34±1.42 | －0.05±0.15 |
| Spinal Cord | 0.01±0.01 | 0.01±0.01 | 0.54±1.03 | －0.30±1.52 | －0.39±1.76 | 1.93±3.40 | 1.20±4.20 | －0.85±2.39 | 0.36±1.82 | 0.00±0.00 |
| MSAP VS. MSMP | | | | | | | | | | |
| Bladder | 0.03±1.74 | －0.03±1.83 | －0.02±0.50 | －0.16±0.59 | －0.17±0.53 | －0.03±0.12 | 0.07±1.46 | 0.06±1.96 | 0.15±3.35 | －0.16±0.45 |
| Rectum | 0.56±1.47 | 0.64±1.80 | 0.24±0.53 | －0.01±0.34 | －0.01±0.32 | 0.04±0.20 | 0.41±1.09 | 0.77±2.09 | 1.00±2.26 | －0.01±0.61 |
| Colon | 0.07±0.33 | 0.04±0.18 | －0.02±0.22 | －0.37±1.17 | －0.35±1.15 | 0.09±1.11 | －0.21±1.29 | －0.02±0.45 | 0.02±0.49 | －0.02±0.10 |
| Femoral Head L | 0.08±0.46 | 0.08±0.42 | 0.10±1.06 | 0.21±1.42 | 0.215±1.48 | 3.42±9.07 | －0.53±4.07 | －1.17±3.49 | －0.19±0.83 | 0.00±0.00 |
| Femoral Head R | －0.01±0.68 | －0.07±0.69 | 0.50±1.03 | 0.91±1.56 | 0.99±1.67 | 1.94±8.61 | 2.15±6.17 | 1.09±1.64 | 0.36±0.68 | 0.00±0.00 |
| Kidney L | －0.01±0.07 | －0.00±0.06 | －0.03±0.18 | －0.12±0.31 | －0.08±0.24 | 0.17±0.37 | 0.01±0.05 | －0.01±0.04 | 0.00±0.00 | 0.00±0.00 |
| Kidney R | 0.05±0.25 | 0.05±0.23 | －0.02±0.06 | －0.04±0.58 | －0.06±0.48 | －0.21±0.48 | 0.01±0.12 | 0.00±0.01 | 0.00±0.00 | 0.00±0.00 |
| Pelvic Bone | 0.17±0.54 | 0.16±0.59 | 0.04±0.20 | 0.03±0.37 | 0.04±0.26 | 0.10±0.37 | 0.35±0.93 | 0.21±1.86 | －0.03±1.36 | 0.01±0.10 |
| Spinal Cord | 0.00±0.01 | 0.00±0.01 | 0.53±0.99 | 0.15±1.18 | 0.20±1.22 | 1.76±3.38 | 1.59±3.07 | 0.41±2.58 | 0.01±0.28 | 0.00±0.00 |

ASAP: Automatic Segmentation in AS-VMAT Plan, MSAP: Manual Segmentation in AS-VMAT Plan, MSMP: Manual Segmentation in MS-VMAT Plan, AS-VMAT: automatic segmentations VMAT, MS-VMAT: manual segmentations VMAT, OARs: organs at risk.
